# Relationship among essentials of magnetism, grit and nursing practice readiness in new graduate nurses: a network analysis

**DOI:** 10.3389/fmed.2026.1784320

**Published:** 2026-07-10

**Authors:** Miaomiao Li, Hongyan Jin, Guiyue Ma, Li Qiao, Yue Wang, Xiang Wang

**Affiliations:** 1 Key Laboratory of Geriatric Nursing and Health, Anhui University of Chinese Medicine, Hefei, Anhui, China; 2 College of Nursing, Catholic University of Pusan, Busan, Republic of Korea

**Keywords:** essentials of magnetism, grit, network analysis, new graduate nurses, practice readiness

## Abstract

**Background:**

The nursing practice readiness of new graduate nurses is a key factor in enabling them to confront transitional challenges, adapt to clinical work, and enhance the quality of nursing care. However, new graduate nurses commonly report a lack of sufficient preparedness upon entering the workforce after graduation.

**Methods:**

Using a cluster sampling method, data were collected from 518 new graduate nurses across 10 hospitals in Central China from April 2025 to September 2025. Network analysis was applied to visualize and identify the core associations among the essentials of magnetism, grit, and nursing practice readiness, and to assess the centrality of each node.

**Results:**

Within the network structure, Clinical judgment and nursing performance, Collaborative interpersonal relationship and Professional attitudes emerged as the most central nodes. Furthermore, Educational Support demonstrated the strongest expected influence, while Collaborative interpersonal relationship and Professional attitudes exhibited the highest bridge strength.

**Conclusion:**

Clinical judgment and nursing performance of new graduate nurses is the most critical node in the network, and Collaborative interpersonal relationship and professional attitudes function as both a central and a bridging nodes. Nursing managers should strengthen educational support and develop targeted intervention programs to enhance nursing practice readiness.

## 1 Introduction

With the rapid development of global healthcare and the accelerating trend of population aging, the demand for clinical nursing services has increased significantly, accompanied by rising expectations for the quality of nursing care. Against this backdrop, healthcare institutions have placed higher expectations on the clinical competence of new graduate nurses, requiring them to adapt swiftly to complex clinical environments and effectively resolve various nursing problems (1).

However, existing studies have shown that new graduate nurses commonly encounter numerous challenges in clinical practice, including insufficient ability to analyze clinical data, difficulty identifying the root causes of problems, low efficiency in integrating information, inaccurate prioritization of nursing issues, and inappropriate selection of interventions (2). These deficiencies not only expose new graduate nurses to significant transitional shocks but also lead to interpersonal tension, anxiety, and professional burnout (3). But may also pose threats to patient safety, as evidenced by higher rates of nursing errors and delayed recovery processes (4). Therefore, to enable new graduate nurses to deliver safer, higher-quality patient care, it is essential that they possess a multidimensional set of competencies and personal attributes.

Practice readiness refers to a new employee’s capacity—including knowledge, skills, and attitudes—to adapt to a specific workplace and respond effectively to various work scenarios and challenges (5). For new graduate nurses, practice readiness extends beyond the mastery of technical skills to encompass critical thinking, clinical judgment, communication skills, professional responsibility, and psychological resilience in coping with the “reality shock” of clinical practice (6, 7). This concept goes beyond traditional assessments of knowledge and skills, emphasizing integrated practical competence within complex clinical environments. Adequate practice readiness enables new nurses to better manage these challenges, adapt more rapidly to new work settings, team cultures, and evolving responsibilities (8). However, influenced by educational and training models, clinical support systems, and individual psychological characteristics, new graduate nurses often experience insufficient practice readiness when transitioning from academic settings to real-world clinical practice (9, 10). Evidence suggests that only approximately 8% of new graduate registered nurses are adequately prepared to perform basic clinical judgments (11). This substantial gap between theory and practice frequently leads to feelings of frustration and stress among new graduate nurses (12), affecting their professional identity and sense of responsibility (8), and may even contribute to nursing workforce attrition (12). This phenomenon has become a shared challenge in global nursing education and management (10), highlighting the need for a systematic investigation into the current status of new graduate nurses’ nursing practice readiness and its influencing factors.

The transition of new graduate nurses from students to clinical practitioners is often influenced by complex, multi-level factors, including personal, interpersonal, and organizational elements, many of which lie beyond their individual control (6). To systematically understand the nursing practice readiness of new graduate nurses, we drew on Albert Bandura’s Triadic Reciprocal Determinism (TRD) (13) as a conceptual sensitizing lens, a theory that has been widely applied to explain nurse behavior and nursing practice (14–16). TRD emphasizes the ongoing, dynamic interactions among the environment, the individual, and behavior. These three components do not exist in isolation or operate in a unidirectional manner; rather, they mutually influence one another, forming a complex interconnected system that collectively shapes individual experiences and actions (13). Within this framework, we represent the environmental determinant with the essentials of magnetism. This concept capturing systemic organizational and cultural support. The individual determinant with grit, which reflects the cognitive perseverance needed to navigate clinical shocks. Viewed through this lens, these two determinants are structurally interconnected with the behavioral outcome, defined as nursing practice readiness, or the actualized capacity to adapt and perform clinical tasks. Grounded in TRD’s core principle that these components operate as an integrated system rather than in isolation (13), this study aims to explore the complex conditional associations among essentials of magnetism, grit, and practice readiness.

In terms of the environment, the concept of the essentials of magnetism originated from the core characteristics of “Magnet Hospitals,” a framework initially operationalized by Kramer and Schmalenberg to identify environments that successfully attract and retain nurses (17). Structurally, this concept encompasses multiple dimensions, including nursing management support, the value of team collaboration, professional autonomy, nurse–physician collaboration, continuing education support, and human resource allocation, providing a systematic measure of a healthcare institution’s attractiveness and organizational support for nursing staff. Magnet hospitals typically offer nurses richer training and learning opportunities, which enhance their professional competence and occupational development, thereby strengthening the readiness of new graduate nurses for nursing practice (18). Empirical studies have further confirmed that essentials of magnetism significantly predict nurse work engagement, quality of care, and psychological wellbeing (19, 20). A study by Xue et al. (21) focusing on new graduate nurses in China found that the essentials of magnetism effectively promote their professional adaptation and significantly reduce turnover intentions. Compared with other organizational environment constructs, essentials of magnetism offer a more comprehensive and systematic framework for evaluating the work environment; however, its specific mechanisms of action in particular contexts still require further research and validation.

Grit is a key concept in positive psychology, defined by Duckworth et al. (22) as “an individual’s sustained passion and perseverance for long-term goals.” Unlike resilience, which is the ability to recover from setbacks, or psychological flexibility, which refers to adapting to changing circumstances, grit uniquely focuses on the persistent, long-term pursuit of personally meaningful goals by combining deep passion with ongoing perseverance. It is more than “stubborn persistence”—it is a structured, value-driven pursuit. As a psychological trait, grit may help new graduate nurses adapt to complex healthcare environments and transition into professional roles (23). Evidence shows that students with higher grit levels perform better in academic and clinical practice, displaying strong goal-directed behavior and professional identity (24). When faced with academic or clinical setbacks, students with high grit are more proactive in seeking solutions (23, 25). Thus, grit may predict how ready new graduate nurses are for practice.

In recent years, network analysis has been successfully applied to the study of nurse psychological fatigue and to the evaluation of nursing education outcomes, revealing the complex interactions between psychological traits and organizational factors (26, 27). By mapping each study variable as a network node, this method can visually present the strength and patterns of associations among variables, as well as identify central nodes and key bridging pathways within the network (28). Although prior studies (19, 21, 23, 24) have examined pairwise relationships, none has integrated all three constructs into a single network analysis to quantify their mutual associations, centralities, and bridging pathways. For new graduate nurses, this analytical approach not only facilitates the identification of key factors influencing practice readiness but also provides a scientific basis for developing multi-level, personalized professional support interventions, thereby promoting a smooth role transition and enhancing both career stability and clinical service competence. In summary, based on Triadic Reciprocal Determinism theory and empirical evidence, this study aims to use network analysis to explore the network structure and interrelationships among essentials of magnetism, grit, and nursing practice readiness in new graduate nurses, offering new data support and empirical insights for workforce development and clinical nursing human resource management.

## 2 Methods

### 2.1 Design and sample

This study is designed as a descriptive cross-sectional survey. This study was reported in strict compliance with the strengthening the reporting of observational studies in epidemiology (STROBE) guideline. Data collection for this study was conducted between April 2025 and September 2025 across 10 hospitals in central China. The participating hospitals included six large general hospitals (with more than 500 beds), one medium-sized hospital (100–400 beds), two community hospitals, and one maternity hospital. The sample size was determined based on the characteristics of network analysis. In this study, 14 nodes were included, yielding 91 potential connections (14 * 13/2). It is generally recommended that each connection be estimated with 3–5 samples (29). Considering an anticipated attrition rate of 10%, the minimum required sample size for this study was calculated to be 506 participants. Inclusion criteria were as follows: (1) age ≥18 years; (2) possession of a valid nursing practice license; (3) employment duration of no more than 1 year. Exclusion criteria were as follows: (1) being on leave (e.g., maternity leave, sick leave, or other long-term leave) at the time of the survey; (2) having a diagnosed psychological disorder or mental illness.

A total of 550 eligible new graduate nurses were invited to complete the electronic questionnaire. Of these, 518 valid responses were received, yielding an effective response rate of 94.18%.

### 2.2 Data collection

Prior to data collection, the researchers visited the participating hospitals. They explained the study’s purpose and methodology to the nursing department directors and obtained permission to collect data. Subsequently, research assistants visited each nursing unit to introduce the study’s objectives and procedures to the unit leaders and new graduate nurses. Recruitment notices were posted on workplace bulletin boards for 14 days. New graduate nurses who voluntarily agreed to participate were required to complete an informed consent form for participation and data use via an online survey platform (SoJump). All data were collected by research personnel who had received standardized training. This ensured data authenticity and research validity. The study was conducted and reported in strict accordance with the STROBE guidelines.

### 2.3 Measurement instruments

#### 2.3.1 General demographic questionnaire

A self-developed questionnaire was used to collect participants’ demographic characteristics, including gender, age, ethnicity, marital status, and educational background.

#### 2.3.2 Essentials of Magnetism (EOM)

The Chinese version of the Essentials of Magnetism scale was developed by Kramer et al. (17) and later translated and validated by Pan et al. (30). This scale was used as a self-report instrument to subjectively assess the perceived level of hospital magnetism. It consists of 45 items, each rated on a five-point Likert scale (1 = strongly disagree to 5 = strongly agree). The scale has seven subscales: nursing manager support (13 items), cultural values (10 items), autonomy in nursing practice (7 items), physician-nurse relationships (4 items), nursing practice management (4 items), educational support (4 items), and rational allocation of nursing human resources (3 items). The total score ranges from 45 to 180. Higher scores indicate a higher perceived level of hospital magnetism among nurses. The scale demonstrates excellent internal consistency, with an overall Cronbach’s *α* coefficient of 0.980. In this study, the Cronbach’s α coefficient was 0.887.

#### 2.3.3 Grit

The Chinese version of the Grit Scale, developed by Duckworth et al. (22) and revised by Zhao et al. (31), was used to assess participants’ grit. The scale consists of 12 items divided into two dimensions: perseverance of effort (6 items) and consistency of interests (6 items). Each item is rated on a five-point Likert scale (1 = strongly disagree to 5 = strongly agree). A total mean score is calculated, with six items reverse-scored. Higher scores indicate greater grit. The scale demonstrated good internal consistency, with a Cronbach’s *α* coefficient of 0.850 in the original validation and 0.910 in this study.

#### 2.3.4 Nursing Practice Readiness Scale (NPRS)

The Chinese version of the Nursing Practice Readiness Scale, originally developed by Kim et al. (32) and recently translated and validated by Zhang et al. (33) in March 2025, was used to assess the nursing practice readiness of newly employed nurses. The scale consists of 35 items across five dimensions: Clinical judgment and nursing performance (16 items), professional attitude (7 items), patient-centeredness (4 items), Self-regulation (3 items), and collaborative interpersonal relationship (5 items). Each item is rated on a four-point Likert scale (1 = strongly disagree to 4 = strongly agree). Higher scores indicate a greater level of nursing practice readiness among new graduate nurses. The scale demonstrated excellent internal consistency, with a Cronbach’s *α* coefficient of 0.951 in the original study and 0.909 in this study.

### 2.4 Data analysis

The data were statistically analyzed using SPSS version 27.0 and R version 4.4.3. To identify associations and core indicators among the essentials of magnetism, grit, and nursing practice readiness, a visual network was constructed using the R package ‘bootnet’ (version 1.6). The network was estimated using the graphical least absolute shrinkage and selection operator (GLASSO) method, implemented via the estimate Network function with the ‘EBICglasso’ default setting (29). Because the network nodes represent dimension total scores rather than individual item scores, the variables were treated as continuous. Accordingly, a Pearson correlation matrix was automatically computed as the input for the network model, with missing data handled by pairwise deletion. Relying on a correlation matrix inherently ensures that the variables are standardized prior to network estimation. Model selection was based on the extended Bayesian information criterion (EBIC), with the tuning hyperparameter gamma set to 0.5 to achieve an optimal balance between identifying true edges and penalizing spurious ones. In this network, the nodes represent observed variables, and the edges reflect statistical associations among them. The thickness and color intensity of the edges indicate the strength of the associations (34).

Subsequently, the centrality and predictability indices of the network nodes were calculated using the R package “qgraph,” including Strength and Expected Influence. Strength reflects the sum of the absolute weights of the edges connecting a given node to all other nodes, which serves to identify the core nodes within the network (28, 35, 36). Expected Influence measures the anticipated impact of a node on other nodes through both direct and indirect connections. A higher average predictability indicates that the network’s internal structure shows stronger mutual predictability, with less variance explained by external factors (37). The R package “networktools” was applied to conduct bridge centrality analysis, which quantifies the extent to which a node connects distinct functional modules. Higher bridge centrality values indicate that the node plays a more crucial role in maintaining information flow across different network dimensions (36).

Finally, to ensure the robustness of the network analysis, this study conducted edge accuracy assessment and centrality stability analysis to evaluate the network model’s accuracy and stability (36). The accuracy of the connections was assessed through 1,000 bootstrap samples, where narrower 95% nonparametric confidence intervals (CIs) indicated greater precision and stability of the estimated edge weights. The stability of the network’s expected influence structure was further examined using case-dropping bootstraps (2,000 iterations). The results were quantified using the correlation stability coefficient (CS-coefficient), with values above 0.25 considered acceptable and values exceeding 0.50 regarded as ideal (36).

## 3 Results

### 3.1 Sociodemographic characteristics of nurses

The nurses had a mean age of 22.38 years (SD = 1.14), ranging from 21 to 26 years, as presented in Table 1, 91.7% were female. 93.1% identified as Han ethnicity, 98.3% of the nurses were unmarried. Regarding educational background, 79.3% held a bachelor’s degree.

**Table 1:** Participant characteristics (*n* = 518).

Variables	*n* / Mean ± SD	%
Age	22.38 ± 1.14	–
Gender		
Female	475	91.7
Male	43	8.3
Marital Status		
Unmarried	509	98.3
Married	9	1.7
Education		
Junior colleges	80	15.4
Undergraduate	411	79.3
Master’s degree	27	5.2
Ethnicity		
Han ethnicity	482	93.1
Ethnic minority	36	6.9

### 3.2 Nursing practice readiness network structure

**Figure 1:**
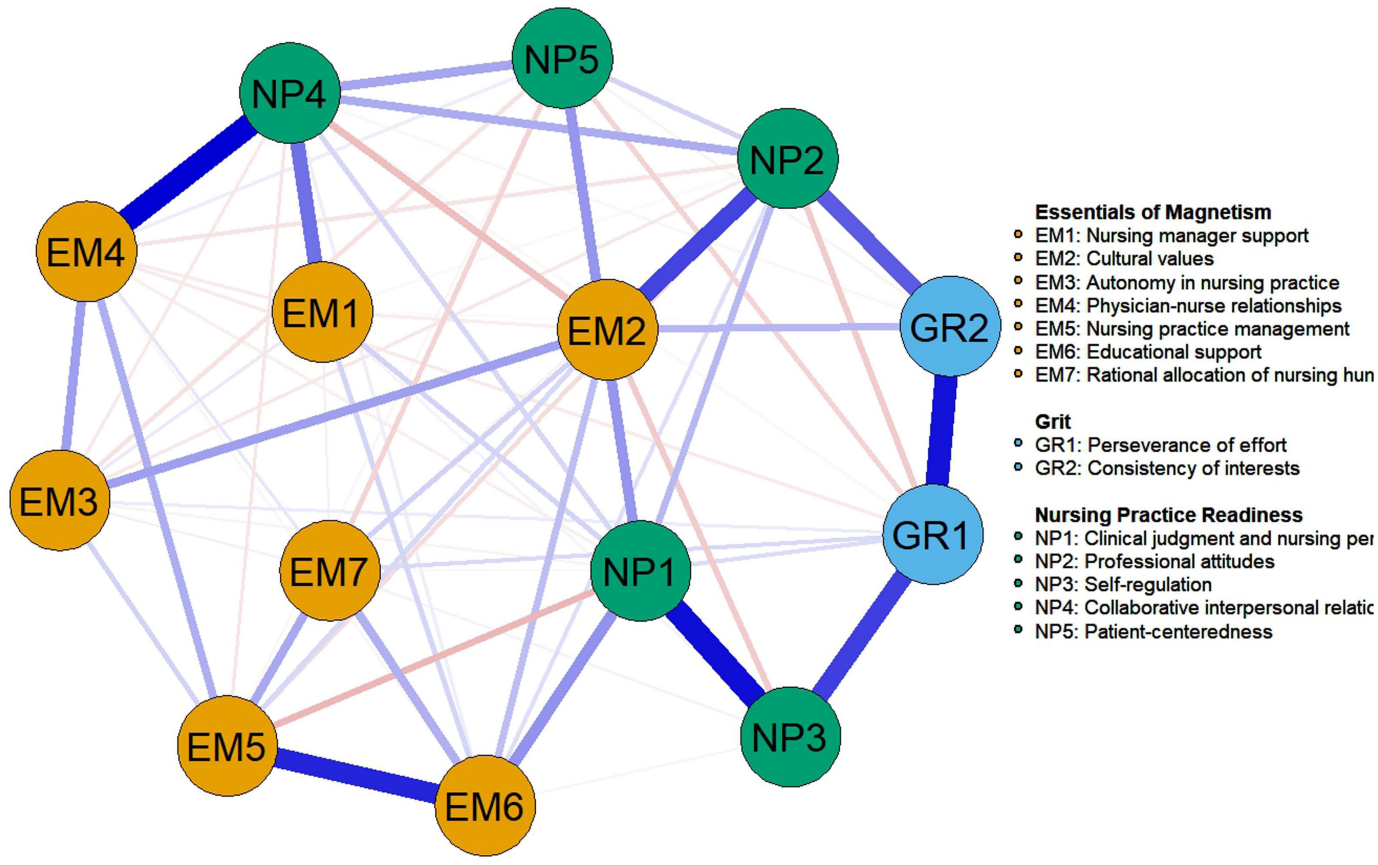
Network structure of essentials of magnetism-grit-nursing practice readiness.

Figure 1 illustrates the network structure of essentials of magnetism–grit–nursing practice readiness among new graduate nurses. The network comprises 14 nodes, each representing one of the 14 dimensions from the three measurement scales. Among the 91 possible connections, 67 (73.6%) exhibited nonzero associations. The three strongest inter-community connections were as follows: NP4 Collaborative interpersonal relationship and EM4 Physician-nurse relationships (weight = 0.590); NP3 Self-regulation and GR1 Perseverance of effort (weight = 0.443); NP2 Professional attitudes and EM2 Cultural values (weight = 0.433). In addition, strong intra-community connections were also observed: NP1 Clinical judgment and nursing performance and NP3 Self-regulation (weight = 0.559); GR1 Perseverance of effort and GR2 Consistency of interests (weight = 0.549); EM5 Nursing practice management and EM6 Educational support (weight = 0.508). All edge weights (association strengths) are listed in Supplementary Table S1, and their bootstrap difference tests are presented in Supplementary Figure S1.

### 3.3 Node centrality and bridge centrality

**Figure 2:**
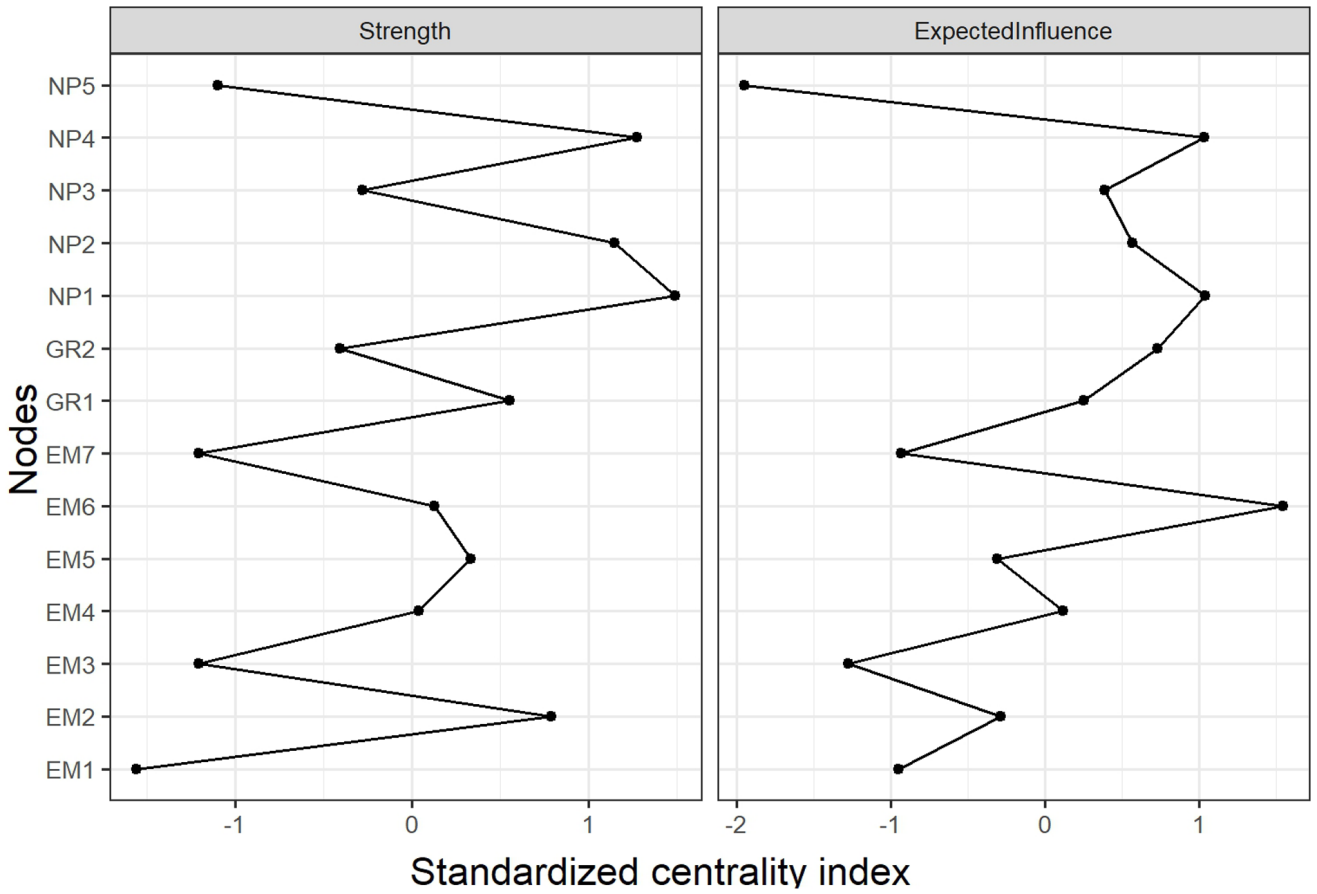
Centrality indicators of all nodes in the network.

**Figure 3:**
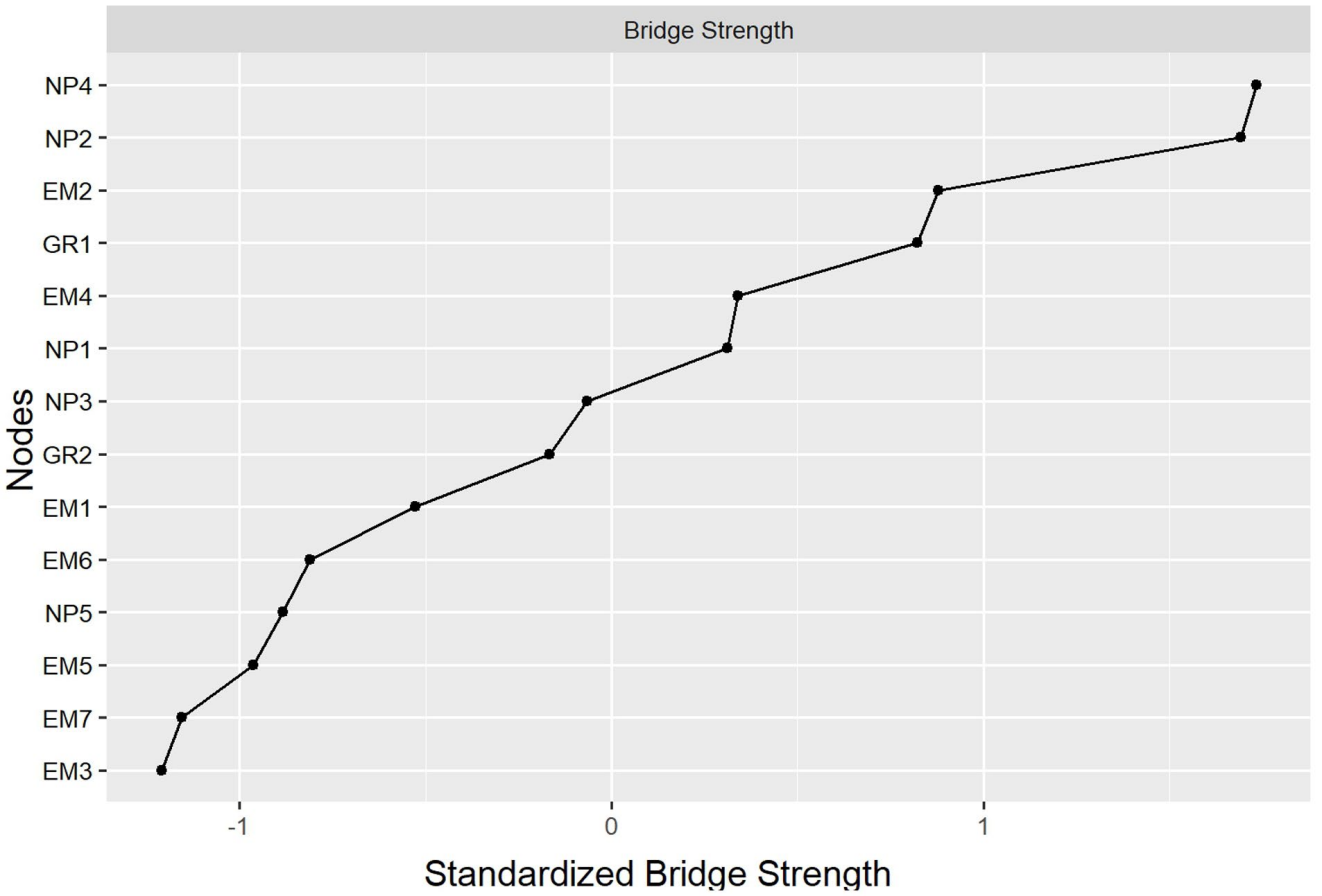
Results of bridge strength analysis.

The study employed strength (S), expected impact (EI), and bridging strength (BS) to assess the importance of each node within the network. The mean values of the variables and the corresponding parameters for each node are shown in Table 2. Regarding strength (Figure 2), NP1 Clinical judgment and nursing performance (*S* = 1.491), NP4 Collaborative interpersonal relationship (*S* = 1.274), NP2 Professional attitudes (*S* = 1.147) exhibited the highest values, indicating that these three nodes served as the most central elements within the network structure. In terms of expected influence, EM6 Educational support (EI = 1.547) had the strongest influence, suggesting that these nodes exert the greatest direct or indirect impact on other nodes within the network. Furthermore, strong intra-community connections were also observed, the strongest edges connected to EM6 included its link with EM5 (weight = 0.508, 95% CI: [0.429, 0.586]) and NP1 (weight = 0.250, 95% CI: [0.180, 0.320]). Bridge strength analysis (Figure 3) revealed that NP4 Collaborative interpersonal relationship (BS = 1.260) and NP2 Professional attitudes (BS = 1.246) showed the highest bridge strength. These findings indicate that these nodes play crucial bridging roles within the network, facilitating connections across different functional communities.

**Table 2:** Centrality indices and predictability of network variables.

Nodes	Strength	Expected influence	Bridge strength
MOE	120.87 ± 9.29		
EM1 Nursing manager support	−1.558	−0.946	0.480
EM2 Cultural values	0.787	−0.286	0.965
EM3 Autonomy in nursing practice	−1.205	−1.274	0.243
EM4 Physician-nurse relationships	0.039	0.122	0.779
EM5 Nursing practice management	0.331	−0.306	0.329
EM6 Educational support	0.130	1.547	0.382
EM7 Rational allocation of nursing human resources	−1.206	−0.932	0.262
Grit	42.83 ± 8.97		
GR1 Perseverance of effort	0.555	0.254	0.945
GR2 Consistency of interests	−0.406	0.734	0.604
NPR	93.31 ± 11.09		
NP1 Clinical judgment and nursing performance	1.491	1.041	0.769
NP2 Professional attitudes	1.147	0.571	1.246
NP3 Self-regulation	−0.281	0.391	0.638
NP4 Collaborative interpersonal relationship	1.274	1.032	1.260
NP5 Patient-centeredness	−1.097	−1.949	0.356

### 3.4 Network stability and accuracy

**Figure 4:**
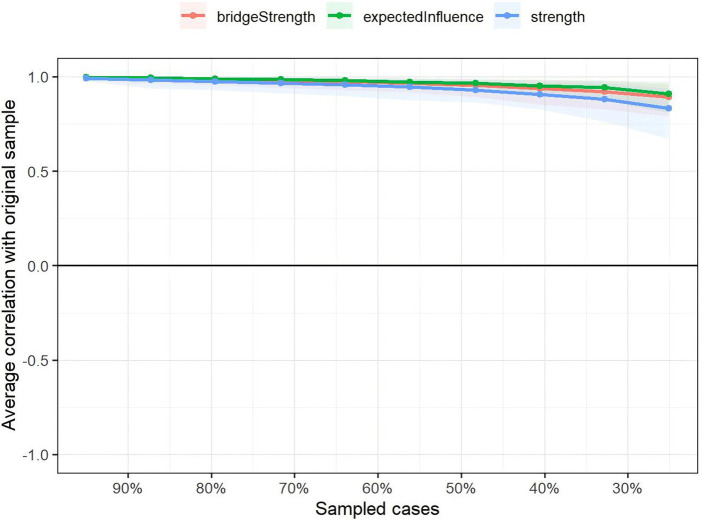
Stability analysis of centrality indices.

**Figure 5:**
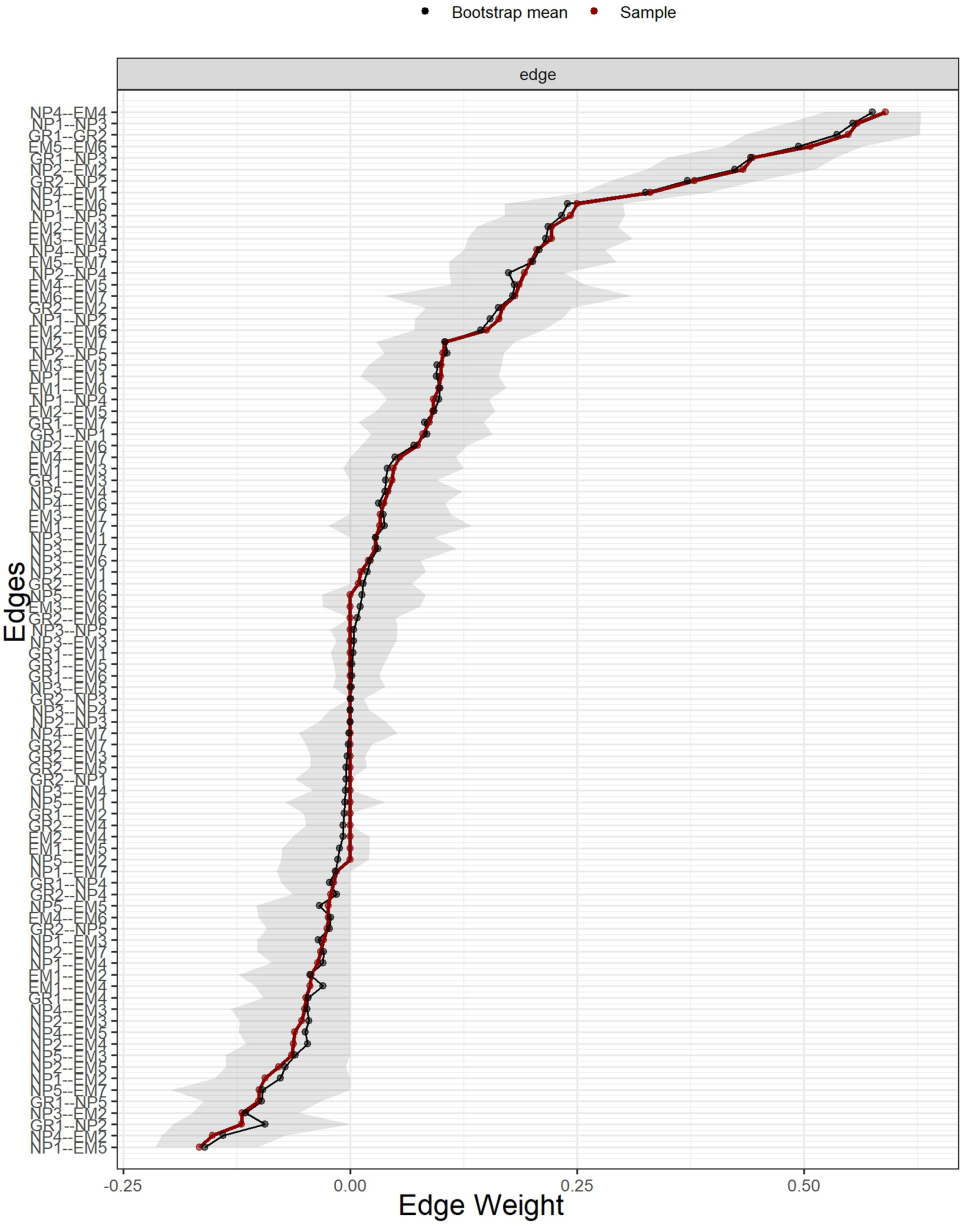
Bootstrap 95% confidence intervals (CIs) for network edge weights.

Figure 4 show the centrality stability analysis conducted for Strength, Expected Influence, and Bridge Strength indices yielded CS-coefficient values of 0.749. These values exceeded the recommended threshold of 0.5, suggesting that the network structure remained robust even with reduced sample sizes. Specifically, the centrality indices retained a high degree of correlation in their rank order and relative magnitude even after the removal of 74.9% of the sample (28). Figure 5 presents the bootstrap 95% confidence intervals (CIs) for edge weights of the network. It can be observed that the bootstrap 95% CIs for most edge weights are generally narrow, demonstrating that the estimated edge weights of the entire network are precise and stable. Detailed numerical values are provided in Supplementary Table S2.

## 4 Discussion

This study adopted Triadic Reciprocal Determinism as a conceptual sensitizing lens and applied network analysis to comprehensively explore the structural relationships among essentials of magnetism, grit, and nursing practice readiness. The study assessed the overall level of nursing practice readiness among new graduate nurses. The findings indicated that the mean score for nursing practice readiness was 93.31 ± 11.09, representing a moderate level. Given this moderate level of readiness, continuous attention and further investigations into the status and influencing mechanisms of nursing practice readiness among new graduate nurses are warranted.

In the nursing practice readiness network model of new graduate nurses, NP1 Clinical judgment and nursing performance, NP4 Collaborative interpersonal relationship, and NP2 Professional attitudes emerged as the core elements of the network, indicating that these nodes play crucial roles in maintaining the connectivity and overall structure of the network. Clinical judgment and nursing performance reflect a nurse’s ability to identify nursing problems, make decisions, and evaluate outcomes in complex clinical situations, as well as to apply acquired knowledge and skills to real-world clinical contexts (38). Within our network model, its high strength centrality indicates that NP1 acts as a “functional core hub” during the professional transition phase of new graduate nurses. While the critical importance of clinical judgment has been widely recognized in previous studies (32, 39), our research further elucidates its systemic role through the lens of network analysis. As the cornerstone for fulfilling clinical responsibilities, NP1 is directly linked to patient safety and health outcomes (38, 40). Consequently, nursing administrators should consider targeting this pivotal node when designing prioritized training and support programs for new graduate nurses. Furthermore, this study also confirmed a close inter-community association between NP1 Clinical judgment and nursing performance and NP3 Self-regulation. This suggests that NP3 could serve as an effective complementary entry point; Previous research (41) has demonstrated that, in clinical practice, self-regulation enables nurses to recognize their own knowledge gaps, actively seek information (e.g., consulting updated clinical guidelines or experienced colleagues), and adjust their understanding and judgment of patients’ conditions based on new evidence. In addition, self-regulation facilitates nurses’ continuous knowledge updating and promotes the effective application of theoretical knowledge in clinical practice, ultimately correlating with superior nursing practice performance (41).

Building upon the critical bridging role of self-regulation (NP3), our network further reveals a profound conditional association with nurses’ internal psychological traits. Specifically, the grit dimension GR1 (Perseverance of effort) exhibits a robust cross-community connection with NP3. As the core dimension of grit, perseverance refers to the behavioral trait of sustained effort and persistence in the face of adversity and failure to achieve long-term goals (42). In clinical settings, nurses consistently endure heavy workloads, emotional exhaustion, and diverse ethical conflicts, all of which rely on sufficient self-regulatory resources for buffering (43, 44). Nurses with high perseverance, even under resource-constrained conditions, can proactively utilize self-regulatory strategies to adapt to job demands, alleviate negative psychological depletion, and actively engage in job crafting. Echoing this bidirectionally, new graduate nurses with outstanding self-regulatory abilities can rely on adjustment mechanisms—such as goal planning, emotional regulation, and cognitive flexibility—to translate their internal drive into sustained behavioral engagement (45). Based on these associative mechanisms, nursing administrators can implement targeted training, such as reflective journaling and periodic feedback, during the clinical preceptorship phase to help new nurses cultivate stable and highly efficient self-regulatory behavioral patterns (46).

NP4 Collaborative interpersonal relationship also occupies a relatively central position within the network, and there is a strong inter-community connection between it and EM4 Physician-nurse relationships. Effective collaboration and communication help new graduate nurses receive timely feedback and support, easing their clinical adaptation and improving judgment and skills (40). Among healthcare interactions, the physician-nurse relationship is especially critical, as it affects patient outcomes, job satisfaction, and system functioning (47). Brown (48) further shows that nurses with positive attitudes toward interprofessional collaboration engage more actively in communication and shared decision-making, thereby optimizing nursing processes and enhancing patient safety and outcomes. Given NP4’s central network position and its strong link to EM4, nurse managers should adopt comprehensive strategies to cultivate a supportive organizational culture, enabling targeted communication within and across departments and disciplines to foster collaborative interpersonal relationships (49).

NP2 Professional attitudes, as a core component of nursing practice readiness, embody nurses’ values, beliefs, codes of conduct, and professional spirit in clinical practice (50), serving as a critical indicator of new graduate nurses’ successful adaptation and development. Beyond confirming its centrality, our network identifies professional attitudes (NP2) as a critical convergence point where internal traits (GR2: Consistency of interests) and external environments (EM2: Cultural values) independently yet simultaneously anchor to shape practice readiness. Consistency of interests, a dimension of grit, stems from intrinsically aligned goals rather than extrinsic motivations (51). Consequently, new graduate nurses sustain enthusiasm when their work resonates deeply with their core identity. The cultural values of a hospital refer to the shared values, beliefs, norms, and behavioral patterns among its members, providing a framework for the hospital’s operations and decision-making processes (52). A hospital culture that emphasizes patient-centered care, teamwork, and the pursuit of excellence can be internalized by nurses, shaping positive professional attitudes (53). For instance, hospitals that promote a culture of safety encourage nurses to develop more positive safety attitudes (54). Recognizing NP2 as a structural nexus, nursing organizations should adopt integrated interventions rather than isolated initiatives. Given that grit is cultivable (51), they may implement individual level programs such as Acceptance and Commitment Therapy (ACT) to help nurses align behaviors with long term goals (55). Simultaneously, at the organizational level, cultivating a positive hospital culture (EM2) is often associated with a supportive work environment, which may help mitigate occupational stress and correlate positively with job satisfaction (56). Integrating these individual and organizational efforts could synergistically promote the development of positive professional attitudes.

EM6 Educational support demonstrated the highest expected influence. While its primary edges’ narrow confidence intervals indicate robust core connections, this metric requires cautious interpretation; as a 4-item subscale, EM6 remains susceptible to regularization artifacts. As one of the essentials of magnetism in hospitals, educational support facilitates the transformation of abstract theoretical knowledge into concrete, practice-integrated skills among new graduate nurses through systematic knowledge updates, skill enhancement, and cultural empowerment (18). Kim et al.’s study (40) found that implementing individualized educational programs for new graduate nurses, such as mentorship training, customized workshops, departmental rotations, preceptorship programs, and peer seminars, can effectively enhance nursing practice readiness. Functioning as a critical reinforcing mechanism, strengthening educational support is likely to generate positive ripple effects throughout the network, helping new graduate nurses bridge the gap between theory and practice. Furthermore, Green et al. (57) showed that high educational support significantly fosters professional values during foundational training. Thus, educational support contributes to readiness for complex clinical environments by providing structured knowledge renewal, reinforcing essential skills, and fostering a supportive, practice-integrated organizational culture.

Bridge strength analysis revealed that NP4 Collaborative interpersonal relationships and NP2 Professional attitudes play a pivotal bridging role within the network, reflecting their importance in maintaining cross-dimensional information flow. Evidence indicates that collaborative interpersonal relationships provide a platform for communication and cooperation among nursing team members (58). New nurses who establish harmonious relationships with colleagues adapt more quickly to workflows, reducing friction and stress in the workplace. Serving as a bridge, collaborative relationships can help new graduate nurses connect theoretical knowledge with practical work through mechanisms such as enhanced mentoring, the provision of relevant resources, and strengthened collaboration (59, 60). Nurses’ professional attitudes can serve as a bridge between their intrinsic values and external behaviors (61). When nurses strongly identify with and internalize professional values, positive professional attitudes facilitate translating these values into concrete nursing actions and behaviors, thereby correlating with the quality of care and patient outcomes. Study (62) conducted in South Korea further demonstrated that professional attitudes exert a significant mediating relationship between nursing students’ critical thinking disposition and their patient safety competence. Therefore, rather than treating these domains as passive descriptive variables, nursing managers can utilize these bridge nodes as precise intervention targets. Cultivating a positive organizational culture and implementing value development programs to strengthen professional attitudes are associated with improvements in overall nursing practice readiness.

Viewed through the theoretical lens of Bandura’s Triadic Reciprocal Determinism (TRD), the findings of this study reveal the complex interconnections among environmental contexts (essentials of magnetism), personal traits (grit), and professional behaviors (nursing practice readiness) in new graduate nurses. This network structure elucidates how they form a mutually reinforcing system. Specifically, professional attitudes (NP2) serve as a cognitive intersection where individual perseverance and organizational culture converge, while collaborative interpersonal relationships (NP4) function as a behavioral bridge connecting the clinical environment with individual clinical judgment. Furthermore, the prominent expected influence of educational support (EM6) underscores its systemic role in activating cognitive resources and facilitating behavioral adaptation. Collectively, these nodes act as dynamic conduits, demonstrating that personal attributes, environmental stimuli, and professional behaviors continuously interact to sustain the nursing practice readiness ecosystem. To facilitate the smooth transition from students to professional nurses, hospitals should implement targeted training centered on these core bridging elements and systemic educational support, leveraging their capacity to create network-wide ripple effects.

## 5 Conclusion

This study innovatively applied network analysis to construct the structural relationships among nursing practice readiness, grit, and the essentials of magnetism in new graduate nurses. The findings revealed that NP1 Clinical judgment and nursing performance, NP4 Collaborative interpersonal relationship, and NP2 Professional attitudes were highly central within the network, with NP4 Collaborative interpersonal relationship and NP2 Professional attitudes demonstrating the strongest bridging effects. This suggests that these nodes are strongly associated with the overall nursing practice readiness of new graduate nurses. Therefore, nursing administrators can develop targeted support programs by focusing on these key nodes to help new graduates adapt more rapidly to clinical practice. Meanwhile, EM6 Educational support displayed the strongest influence, indicating that hospitals should place greater emphasis on educational support, highlighting its potential value as a correlate of nursing practice readiness in new graduate nurses.

### 5.1 Limitations

This study has several limitations. First, the cross-sectional design precludes causal inferences; the network edges reflect conditional associations rather than directional pathways, and centrality indicates interconnectedness. Furthermore, applying network analysis to psychometric data requires acknowledging that some edges may partially reflect shared variance from unmeasured latent constructs rather than pure mechanistic interactions (63). Methodologically, GLASSO regularization may inadvertently shrink small true associations to zero (i.e., regularization bias), and estimation stability depends heavily on sample size. Future longitudinal studies with larger cohorts are warranted to establish causality and solidify structural stability. Second, relying solely on self-reports may introduce response bias, highlighting the need for future research incorporating behavioral assessments for data triangulation. Third, utilizing dimension-level scores (14 nodes) instead of item-level data collapses within-subscale covariation, potentially inflating edge weights. Additionally, structural asymmetry may disproportionately inflate the centrality of larger dimensions. Future research should employ item-level or latent network modeling. Finally, recruiting exclusively from Central China restricts generalizability. Future research should therefore adopt diverse sampling strategies across different regions to enhance external validity.

## Data Availability

The raw data supporting the conclusions of this article will be made available by the authors, without undue reservation.
