# Correction: Relationship among essentials of magnetism, grit and nursing practice readiness in new graduate nurses: a network analysis

**DOI:** 10.3389/fmed.2026.1934247

**Published:** 2026-07-23

**Authors:** Miaomiao Li, Hongyan Jin, Guiyue Ma, Li Qiao, Yue Wang, Xiang Wang

**Affiliations:** 1 Key Laboratory of Geriatric Nursing and Health, Anhui University of Chinese Medicine, Hefei, Anhui, China; 2 College of Nursing, Catholic University of Pusan, Busan, Republic of Korea

**Keywords:** essentials of magnetism, grit, network analysis, new graduate nurses, practice readiness

There was an error in Table 1 as published. The data for “Gender” and “Marital status” were transposed due to a formatting oversight. The corrected Table 1 appears below:

**Table 1:** Participant characteristics (*n* = 518).

Variables	n/Mean ±SD	%
Age	22.38 ± 1.14	–
Gender		
Female	475	91.7
Male	43	8.3
Marital status		
Unmarried	509	98.3
Married	9	1.7
Education		
Junior colleges	80	15.4
Undergraduate	411	79.3
Master's degree	27	5.2
Ethnicity		
Han ethnicity	482	93.1
Ethnic minority	36	6.9

The original version of this article has been updated.

