# Supplementary material for: Relationship among essentials of magnetism, grit and nursing practice readiness in new graduate nurses: a network analysis
Source: Front Med (Lausanne). 2026 Jul 10;13:1784320. doi: 10.3389/fmed.2026.1784320 (PMC13446270; doi:10.3389/fmed.2026.1784320)
Supplement: Supplementary file 1 [file Table_1.DOCX]

Supplementary Material

# 1 Supplementary Figures and Tables

## 1.1 Supplementary Figures

**Supplementary Figure 1.**  **Bootstrap difference tests of the edge weights**

**Note:** Gray boxes indicate no significant difference in edge weights, while black boxes denote a significant difference. The blue and red boxes along the diagonal correspond to positively and negatively correlated edge weights, respectively

## 1.2 Supplementary Tables

## Supplementary Tables 1. Weights of each connection in the network of essentials of magnetism-grit-nursing practice readiness(n=518)

|  | EM1 | EM2 | EM3 | EM4 | EM5 | EM6 | EM7 | GR1 | GR2 | NP1 | NP2 | NP3 | NP4 | NP5 |
| --- | --- | --- | --- | --- | --- | --- | --- | --- | --- | --- | --- | --- | --- | --- |
| EM1 | 1.000 |  |  |  |  |  |  |  |  |  |  |  |  |  |
| EM2 | -0.043 | 1.000 |  |  |  |  |  |  |  |  |  |  |  |  |
| EM3 | 0.048 | 0.222 | 1.000 |  |  |  |  |  |  |  |  |  |  |  |
| EM4 | -0.044 | 0.000 | 0.222 | 1.000 |  |  |  |  |  |  |  |  |  |  |
| EM5 | 0.000 | 0.091 | 0.101 | 0.187 | 1.000 |  |  |  |  |  |  |  |  |  |
| EM6 | 0.097 | 0.150 | 0.000 | -0.024 | 0.508 | 1.000 |  |  |  |  |  |  |  |  |
| EM7 | 0.032 | 0.105 | 0.033 | 0.055 | 0.199 | 0.182 | 1.000 |  |  |  |  |  |  |  |
| GR1 | 0.000 | 0.000 | 0.046 | -0.049 | 0.000 | 0.000 | 0.087 | 1.000 |  |  |  |  |  |  |
| GR2 | 0.009 | 0.168 | 0.000 | 0.000 | 0.000 | 0.000 | 0.000 | 0.549 | 1.000 |  |  |  |  |  |
| NP1 | 0.100 | -0.094 | -0.029 | -0.036 | -0.166 | 0.250 | -0.015 | 0.080 | 0.000 | 1.000 |  |  |  |  |
| NP2 | 0.012 | 0.433 | -0.053 | -0.062 | -0.079 | 0.074 | -0.032 | -0.120 | 0.380 | 0.164 | 1.000 |  |  |  |
| NP3 | 0.028 | -0.119 | 0.000 | 0.000 | 0.000 | 0.020 | 0.028 | 0.443 | 0.000 | 0.559 | 0.000 | 1.000 |  |  |
| NP4 | 0.331 | -0.151 | -0.050 | 0.590 | -0.061 | 0.037 | 0.000 | -0.018 | -0.022 | 0.091 | 0.192 | 0.000 | 1.000 |  |
| NP5 | 0.000 | 0.000 | -0.064 | 0.042 | -0.023 | 0.000 | -0.100 | -0.101 | -0.026 | 0.243 | 0.102 | 0.000 | 0.206 | 1.000 |

## Supplementary Tables 2. Bootstrap 95% confidence intervals for all edge weights in the network(n=518)

| edge | weight | mean | SD | CI lower | CI upper |
| --- | --- | --- | --- | --- | --- |
| NP4--EM4 | 0.590 | 0.576 | 0.027 | 0.537 | 0.644 |
| NP1--NP3 | 0.559 | 0.553 | 0.039 | 0.480 | 0.637 |
| GR1--GR2 | 0.549 | 0.535 | 0.048 | 0.453 | 0.645 |
| EM5--EM6 | 0.508 | 0.493 | 0.039 | 0.429 | 0.586 |
| GR1--NP3 | 0.444 | 0.444 | 0.050 | 0.344 | 0.543 |
| NP2--EM2 | 0.433 | 0.423 | 0.050 | 0.333 | 0.534 |
| GR2--NP2 | 0.380 | 0.369 | 0.042 | 0.295 | 0.464 |
| NP4--EM1 | 0.331 | 0.324 | 0.035 | 0.260 | 0.401 |
| NP1--EM6 | 0.250 | 0.239 | 0.035 | 0.180 | 0.320 |
| NP1--NP5 | 0.243 | 0.232 | 0.031 | 0.181 | 0.305 |
| EM2--EM3 | 0.222 | 0.217 | 0.041 | 0.140 | 0.305 |
| EM3--EM4 | 0.222 | 0.214 | 0.046 | 0.130 | 0.314 |
| NP4--NP5 | 0.206 | 0.209 | 0.038 | 0.129 | 0.283 |
| EM5--EM7 | 0.199 | 0.202 | 0.046 | 0.107 | 0.292 |
| NP2--NP4 | 0.192 | 0.174 | 0.033 | 0.127 | 0.257 |
| EM4--EM5 | 0.187 | 0.182 | 0.037 | 0.114 | 0.260 |
| EM6--EM7 | 0.182 | 0.178 | 0.069 | 0.043 | 0.320 |
| GR2--EM2 | 0.168 | 0.164 | 0.043 | 0.082 | 0.253 |
| NP1--EM5 | -0.166 | -0.160 | 0.028 | -0.222 | -0.110 |
| NP1--NP2 | 0.164 | 0.155 | 0.042 | 0.079 | 0.248 |
| NP4--EM2 | -0.151 | -0.140 | 0.034 | -0.220 | -0.083 |
| EM2--EM6 | 0.150 | 0.145 | 0.036 | 0.079 | 0.222 |
| GR1--NP2 | -0.120 | -0.091 | 0.050 | -0.221 | -0.019 |
| NP3--EM2 | -0.119 | -0.115 | 0.030 | -0.179 | -0.059 |
| EM2--EM7 | 0.105 | 0.104 | 0.038 | 0.029 | 0.180 |
| NP2--NP5 | 0.102 | 0.106 | 0.033 | 0.035 | 0.169 |
| EM3--EM5 | 0.101 | 0.097 | 0.038 | 0.024 | 0.177 |
| GR1--NP5 | -0.101 | -0.099 | 0.030 | -0.161 | -0.040 |
| NP5--EM7 | -0.100 | -0.097 | 0.050 | -0.201 | 0.001 |
| NP1--EM1 | 0.100 | 0.095 | 0.037 | 0.025 | 0.174 |
| EM1--EM6 | 0.097 | 0.100 | 0.035 | 0.028 | 0.167 |
| NP1--EM2 | -0.094 | -0.077 | 0.036 | -0.167 | -0.021 |
| NP1--NP4 | 0.092 | 0.097 | 0.027 | 0.037 | 0.146 |
| EM2--EM5 | 0.091 | 0.093 | 0.034 | 0.024 | 0.159 |
| GR1--EM7 | 0.087 | 0.080 | 0.036 | 0.015 | 0.160 |
| GR1--NP1 | 0.080 | 0.084 | 0.035 | 0.010 | 0.150 |
| NP2--EM5 | -0.079 | -0.072 | 0.034 | -0.146 | -0.011 |
| NP2--EM6 | 0.074 | 0.070 | 0.031 | 0.012 | 0.137 |
| NP5--EM3 | -0.064 | -0.061 | 0.037 | -0.138 | 0.009 |
| NP2--EM4 | -0.062 | -0.046 | 0.031 | -0.124 | -0.001 |
| NP4--EM5 | -0.061 | -0.048 | 0.033 | -0.128 | 0.006 |
| EM4--EM7 | 0.055 | 0.048 | 0.033 | -0.010 | 0.120 |
| NP2--EM3 | -0.053 | -0.047 | 0.035 | -0.124 | 0.018 |
| NP4--EM3 | -0.050 | -0.048 | 0.040 | -0.130 | 0.030 |
| GR1--EM4 | -0.049 | -0.046 | 0.025 | -0.099 | 0.002 |
| EM1--EM3 | 0.048 | 0.042 | 0.038 | -0.029 | 0.124 |
| GR1--EM3 | 0.046 | 0.038 | 0.027 | -0.007 | 0.099 |
| EM1--EM4 | -0.044 | -0.030 | 0.033 | -0.110 | 0.022 |
| EM1--EM2 | -0.043 | -0.044 | 0.035 | -0.112 | 0.027 |
| NP5--EM4 | 0.042 | 0.037 | 0.035 | -0.028 | 0.112 |
| NP4--EM6 | 0.037 | 0.032 | 0.030 | -0.023 | 0.097 |
| NP1--EM4 | -0.036 | -0.029 | 0.026 | -0.088 | 0.017 |
| EM3--EM7 | 0.033 | 0.036 | 0.035 | -0.036 | 0.102 |
| EM1--EM7 | 0.032 | 0.037 | 0.040 | -0.048 | 0.112 |
| NP2--EM7 | -0.032 | -0.028 | 0.030 | -0.092 | 0.028 |
| NP1--EM3 | -0.029 | -0.031 | 0.029 | -0.087 | 0.029 |
| NP3--EM1 | 0.028 | 0.029 | 0.029 | -0.030 | 0.086 |
| NP3--EM7 | 0.028 | 0.031 | 0.035 | -0.043 | 0.098 |
| GR2--NP5 | -0.026 | -0.020 | 0.024 | -0.074 | 0.023 |
| EM4--EM6 | -0.024 | -0.021 | 0.030 | -0.084 | 0.036 |
| NP5--EM5 | -0.024 | -0.033 | 0.031 | -0.085 | 0.037 |
| GR2--NP4 | -0.022 | -0.014 | 0.019 | -0.060 | 0.016 |
| NP3--EM6 | 0.020 | 0.022 | 0.024 | -0.027 | 0.067 |
| GR1--NP4 | -0.018 | -0.022 | 0.023 | -0.064 | 0.028 |
| NP1--EM7 | -0.015 | -0.015 | 0.025 | -0.064 | 0.035 |
| NP2--EM1 | 0.012 | 0.019 | 0.026 | -0.039 | 0.063 |
| GR2--EM1 | 0.009 | 0.013 | 0.020 | -0.030 | 0.049 |
| EM1--EM5 | 0.000 | -0.013 | 0.023 | -0.046 | 0.046 |
| EM2--EM4 | 0.000 | -0.009 | 0.020 | -0.039 | 0.039 |
| EM3--EM6 | 0.000 | 0.010 | 0.023 | -0.045 | 0.045 |
| GR1--EM1 | 0.000 | 0.003 | 0.013 | -0.025 | 0.025 |
| GR1--EM2 | 0.000 | -0.008 | 0.017 | -0.033 | 0.033 |
| GR1--EM5 | 0.000 | 0.003 | 0.010 | -0.020 | 0.020 |
| GR1--EM6 | 0.000 | 0.003 | 0.012 | -0.024 | 0.024 |
| GR2--EM3 | 0.000 | -0.004 | 0.015 | -0.030 | 0.030 |
| GR2--EM4 | 0.000 | -0.007 | 0.015 | -0.029 | 0.029 |
| GR2--EM5 | 0.000 | -0.004 | 0.014 | -0.027 | 0.027 |
| GR2--EM6 | 0.000 | 0.009 | 0.016 | -0.032 | 0.032 |
| GR2--EM7 | 0.000 | -0.001 | 0.015 | -0.030 | 0.030 |
| GR2--NP1 | 0.000 | -0.005 | 0.016 | -0.033 | 0.033 |
| GR2--NP3 | 0.000 | 0.000 | 0.007 | -0.015 | 0.015 |
| NP2--NP3 | 0.000 | 0.000 | 0.013 | -0.026 | 0.026 |
| NP3--EM3 | 0.000 | 0.003 | 0.015 | -0.030 | 0.030 |
| NP3--EM4 | 0.000 | -0.005 | 0.012 | -0.024 | 0.024 |
| NP3--EM5 | 0.000 | 0.001 | 0.011 | -0.022 | 0.022 |
| NP3--NP4 | 0.000 | 0.000 | 0.011 | -0.022 | 0.022 |
| NP3--NP5 | 0.000 | 0.006 | 0.018 | -0.035 | 0.035 |
| NP4--EM7 | 0.000 | 0.000 | 0.020 | -0.040 | 0.040 |
| NP5--EM1 | 0.000 | -0.004 | 0.023 | -0.046 | 0.046 |
| NP5--EM2 | 0.000 | -0.014 | 0.023 | -0.046 | 0.046 |
| NP5--EM6 | 0.000 | 0.013 | 0.027 | -0.054 | 0.054 |
